# Supplementary material for: Ecoresorbable chipless temperature-responsive tag made from biodegradable materials for sustainable IoT
Source: Nat Commun. 2025 Nov 25;16:10478. doi: 10.1038/s41467-025-65458-9 (PMC12647737; doi:10.1038/s41467-025-65458-9)
Supplement: Supplementary file 1 — Supplementary Information [file 41467_2025_65458_MOESM1_ESM.pdf]

Supplementary information

Ecoresorbable chipless temperature-responsive tag made from biodegradable materials for sustainable IoT

James Bourely[1], Nicolas Fumeaux[1], Xavier Aeby[2], Jaemin Kim[1], Gilberto Siqueira[2], Christian Beyer[3], David Schmid[3], Oleksandr Vorobyov[3], Gustav Nyström[2,4]* and Danick Briand[1]**

[1] Ecole Polytechnique Fédérale de Lausanne (EPFL), Soft Transducers Laboratory (LMTS), Rue de la Maladière 71b, 2002 Neuchâtel, Switzerland. E-mail: james.bourely@epfl.ch, danick.briand@epfl.ch

[2] Swiss Federal Laboratories for Materials Science and Technology (Empa), Cellulose & Wood Materials Laboratory, Überlandstrasse 129, 8600 Dübendorf, Switzerland

[3] CSEM SA, Rue Jaquet-Droz 1, 2002 Neuchâtel, Switzerland

[4] ETH Zürich, Department of Health Science and Technology, 8092 Zürich, Switzerland. E-mail: gustav.nystroem@empa.ch

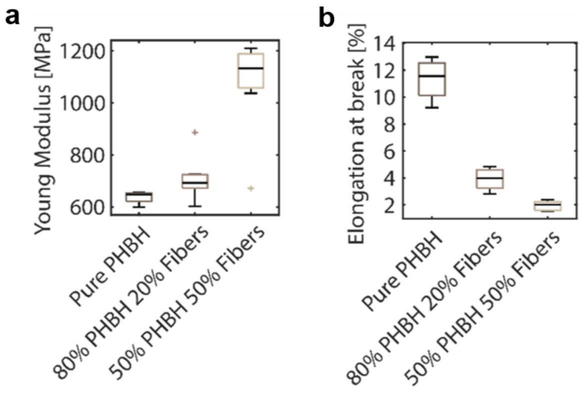

Fig. S1 | Young's modulus and elongation at break of the pure PHBH and its cellulose composites. **a** Young's Modulus for 0 wt%, 20 wt% and 50 wt% of cellulose fibers in PHBH. **b** Elongation at break for 0 wt%, 20 wt% and 50 wt% of cellulose fibers in PHBH.

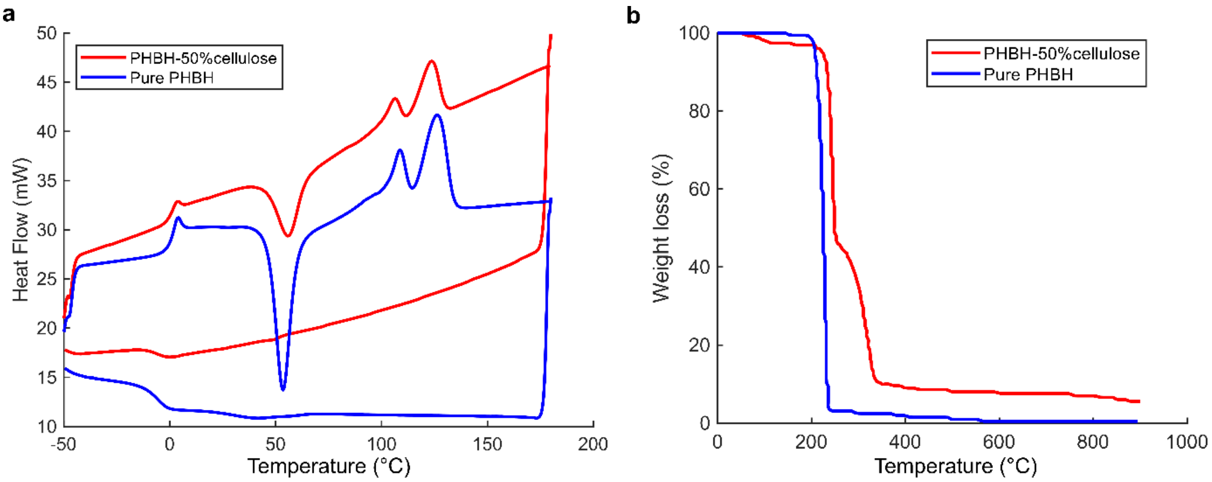

Fig. S2 | Thermal analysis of the pure PHBH and its cellulose 50% composite. **a** Differential scanning calorimeter measurement from -50 to 200 °C for pure PHBH and 50%cellulose composite, 15 mg samples. **b** Thermogravimetric analysis of pure PHBH and PHBH-50%cellulose.

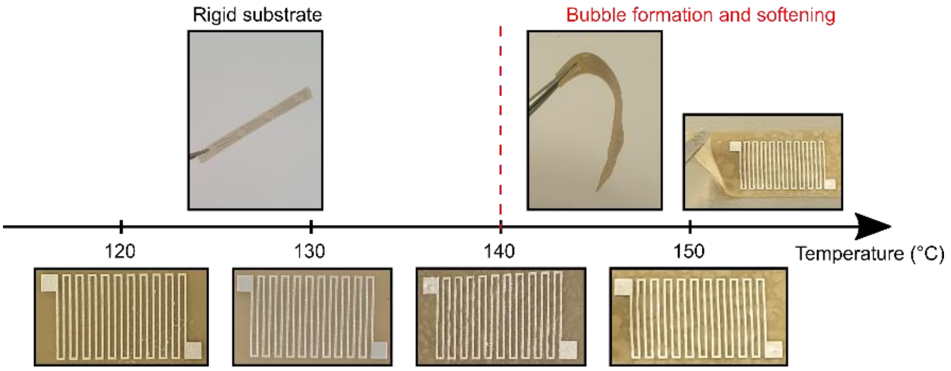

Fig. S3 | Thermal stability of PHBH-50%cellulose with silver printed serpentine. Sintering in the oven for 30 min at 120, 130, 140, 150 with optical images of the printed silver resistors. After 140 °C sintering, the PHBH-50%cellulose composite loses its stiffness and bubbles appear.

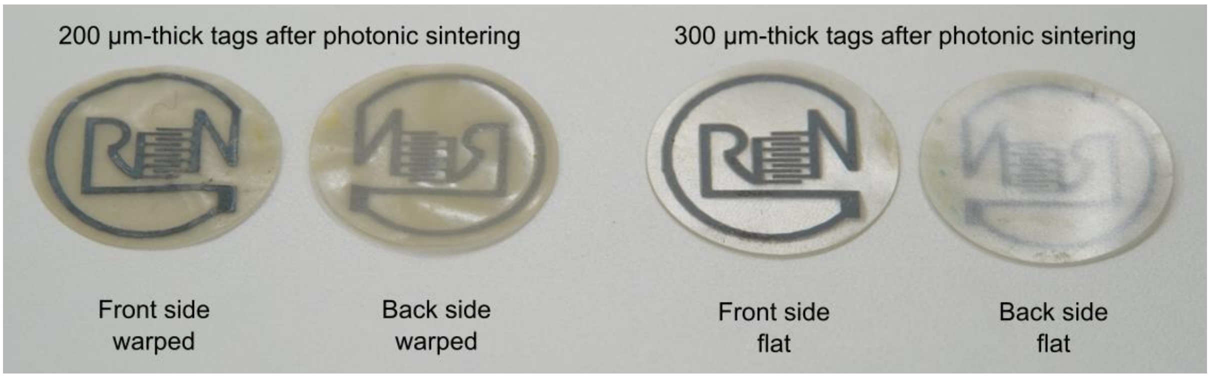

Fig. S4 | Warping of the 200 µm-thick substrates after photonic sintering of the zinc layer as opposed to the 300 µm-thick substrates. Optical images showing 4 different tags after photonic sintering of the zinc printed layer. On the left, wrinkles due to warping can be seen on the front and back side of 200 µm-thick devices. On the right, the 300 µm-thick tags do not show any signs of warping after sintering.

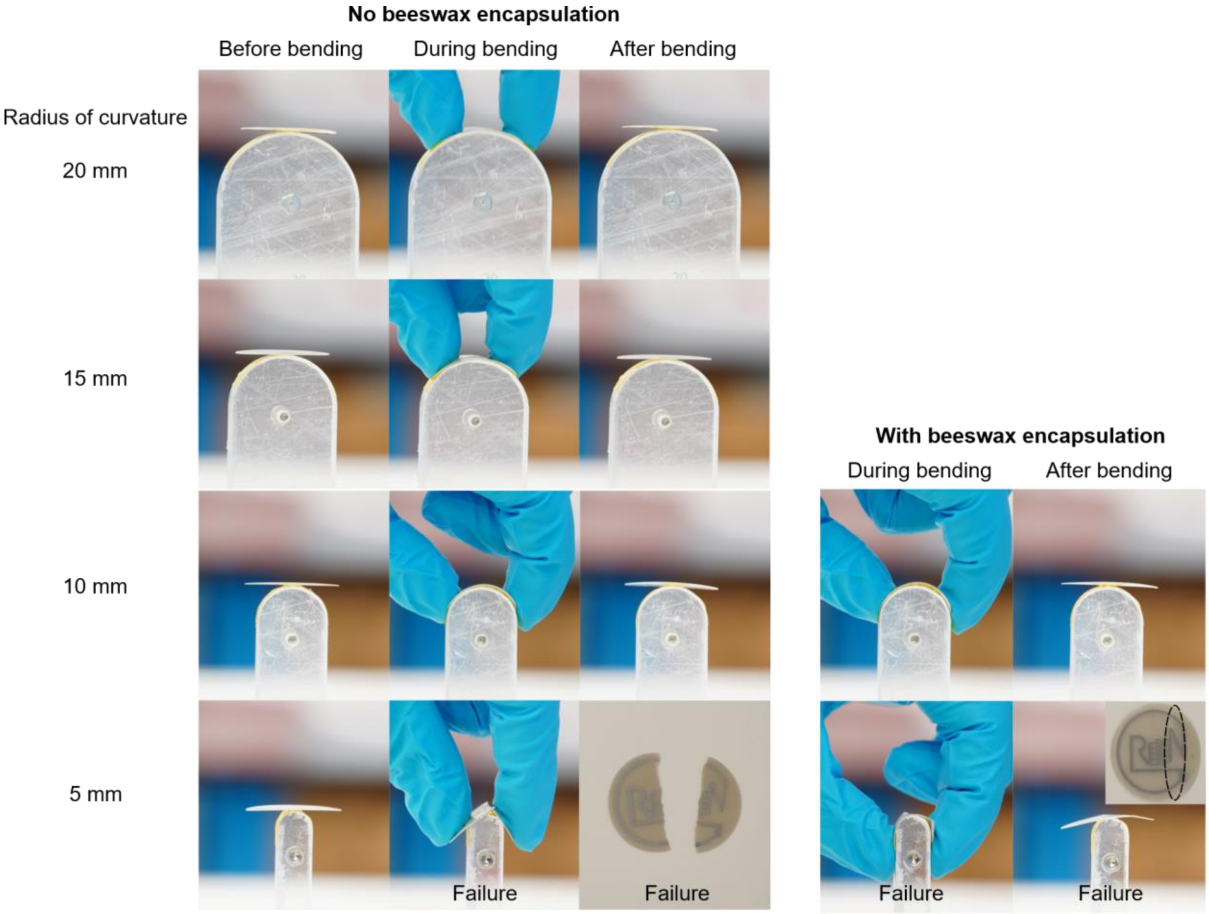

Fig. S5 | Bending of the tag with and without beeswax encapsulation over different radii of curvature. Optical images showing the tag, before, during and after being bent over structures having 20, 15, 10 and 5 mm radii of curvature. The 300 µm-thick tag fails when bent over a 5 mm radius, regardless of the presence of beeswax. The 100 µm-thick beeswax encapsulation layer prevented the tag from breaking into two pieces, despite the failure of the PHBH-composite substrate.

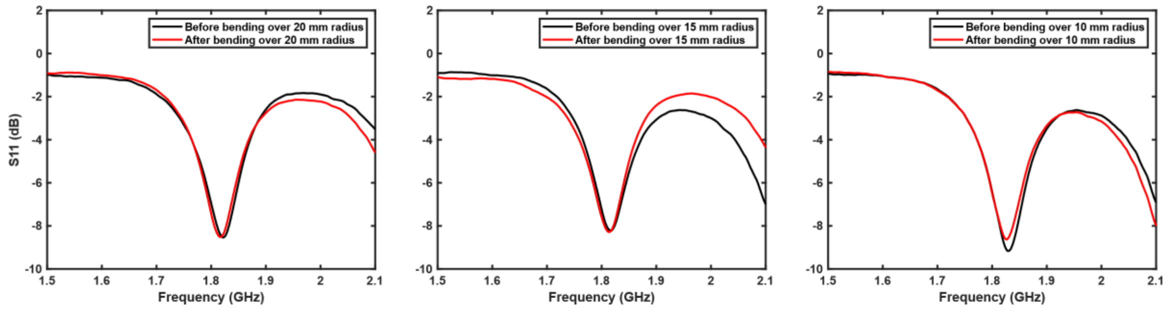

Fig. S6 | S_{11} response of the tag with beeswax encapsulation before and after bending at 20, 15 and 10 mm radius of curvature. The frequency of resonance of the pristine tag after bending shifted by only 3 MHz for all cases.

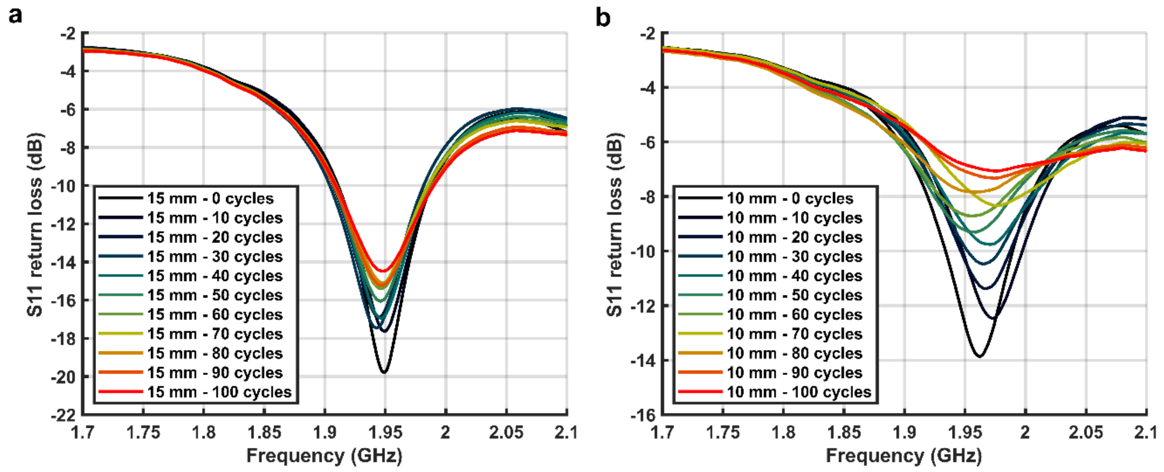

Fig. S7 | Evolution of the S_{11} response of the tag with beeswax encapsulation after 100 bending cycles. a at 15 mm radius of curvature and **b** at 10 mm radius of curvature.

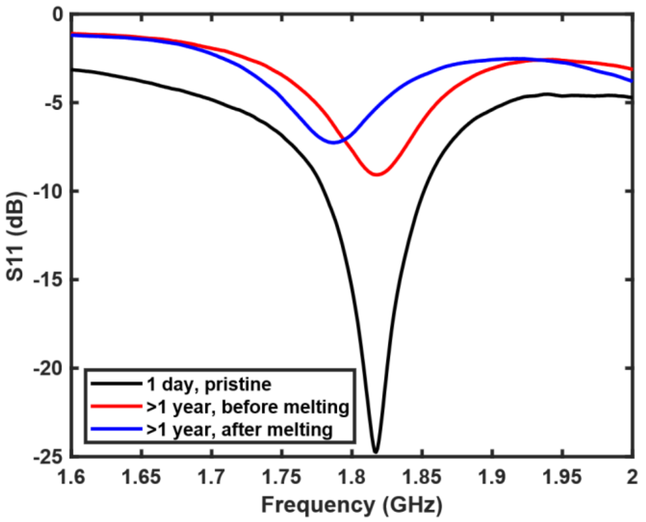

Fig. S8 | Response of the printed chipless tag after 1 year at ambient conditions. The frequency of resonance of the tag with beeswax, after fabrication and 1 year later. The frequency of resonance of the encapsulated tags exhibits a drift of only 4 MHz after 1 year with an amplitude drop of 16 dB. After melting of the PCM, the 1-year-old tag still provided a resonance shift of 34 MHz.

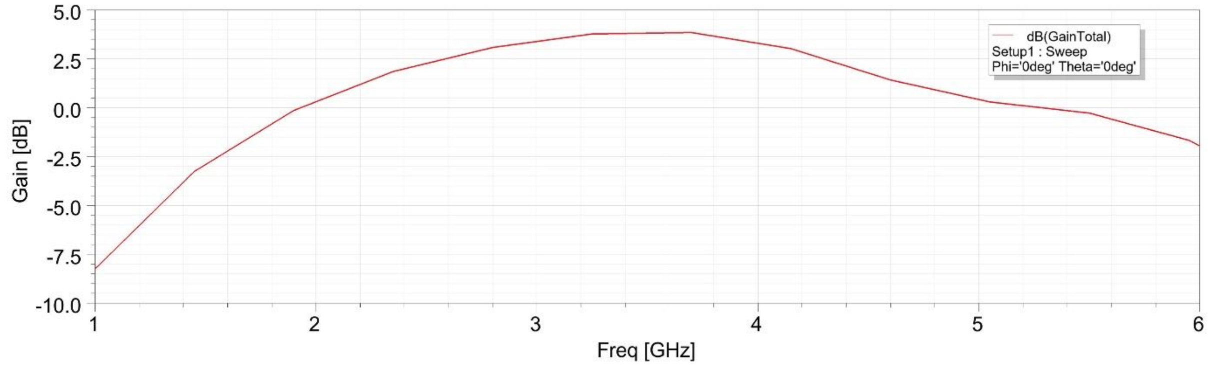

Fig. S9 | Simulated antenna gain as a function of the frequency from 1 to 6 GHz. The relatively smooth curve indicates stable directional performance across the frequency range.

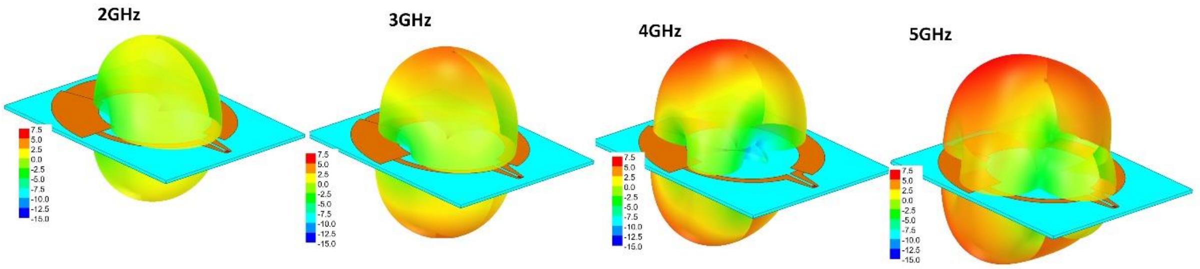

Fig. S10 | Antenna 3D radiation pattern at different frequencies. Gain is presented at 2, 3, 4 and 5 GHz.

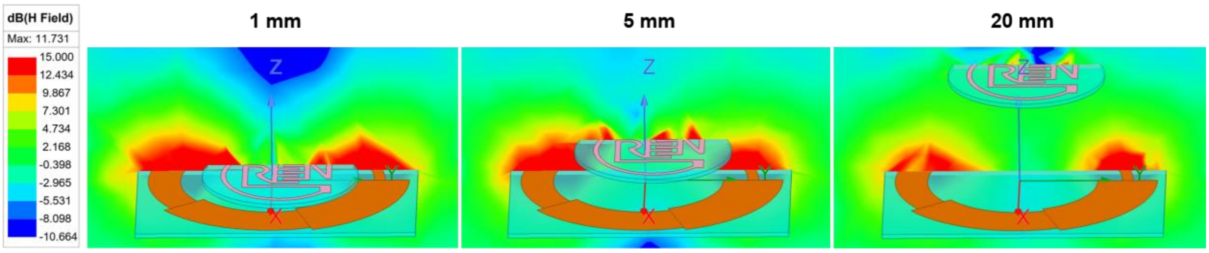

Fig. S11 | The antenna and passive tag coupling theoretical visualisation at 1, 5 and 20 mm separation distances. At 20 mm distance, the tag is decoupled from the antenna.

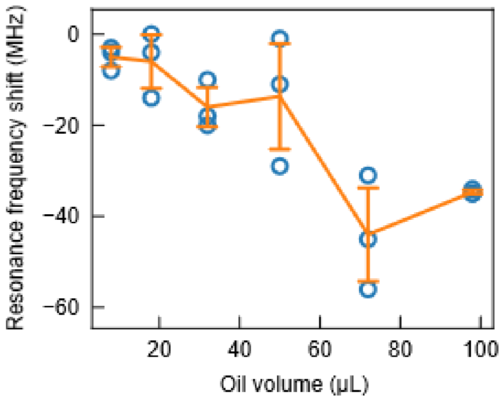

Fig. S12 | Frequency shift of the tag after melting of a 2 mm-thick frozen coconut oil. Area of the frozen PCM cube over the IDEs is varying from 2x2 to 7x7 mm^2 corresponding to oil volumes of 8 to 98 μL.

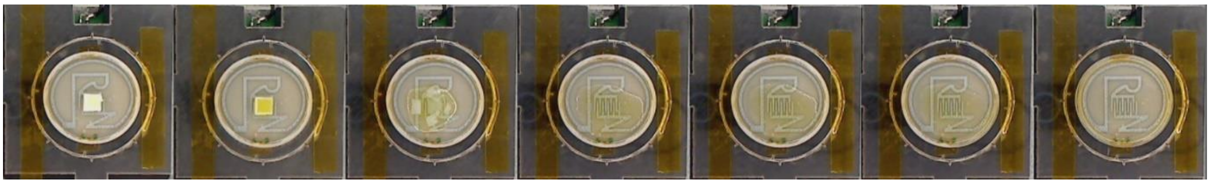

Fig. S13 | Evolution of the spreading of the olive oil over time. The 144 µL olive oil cube melts as the temperature is increased from 0 °C to 30 °C. At higher temperatures, when the liquid oil reaches the edge of the tag, it can be seen seeping between the tag and the reader antenna. This results in an undesired modification of the dielectric properties of the tag and explains the second drop in frequency of resonance of the olive oil-based tag in **Fig. 3f**.

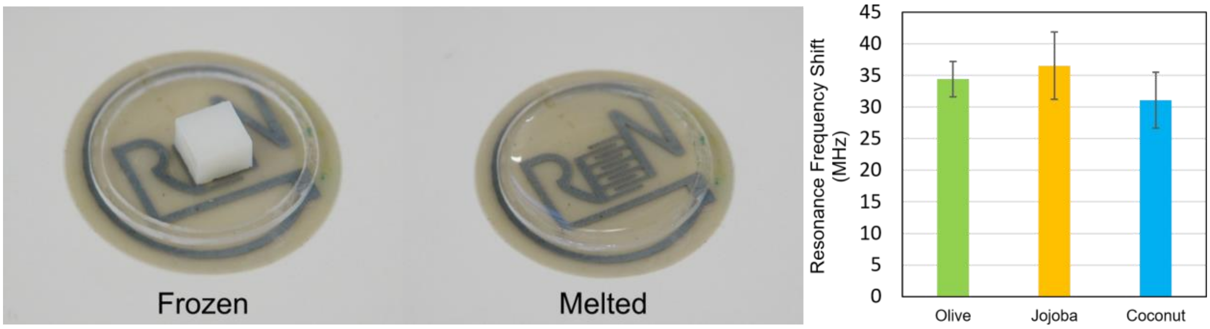

Fig. S14 | Confining the melted PCM over the tag using a plastic well leads to similar resonance frequency shifts for all three oils. The 144 µL oil cube melts as the temperature is increased from 0 °C to 30 °C with a plastic well used to confine the oil over the tag. The measured resonance frequency shift for the olive, jojoba and coconut oils are presented on the right and are $34.4 \pm 2.8\ MHz, 36.5 \pm 5.3\ MHz$ and $31.1 \pm 4.4\ MHz$, respectively (n = 3).

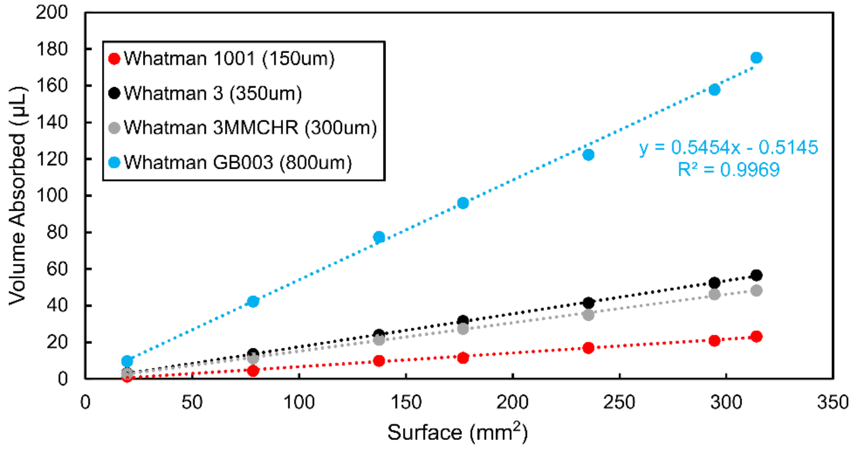

Fig. S15 | Absorption capacity of commercially available cellulose filter elements based on their surface area. Liquid jojoba oil at room temperature is soaked in laser cut paper filters disk ranging from 20 mm^2 to 314 mm^2 in size. Maximum volume absorbed is calculated using the density of jojoba oil (0.87 g/mL). We have the following equation for the selected 100% cellulose Whatman GB003 absorbent: $Maximum\ oil\ mass\ absorbed\ [mg] = \frac{0.54}{0.87} * area\ of\ paper\ [mm^2]$

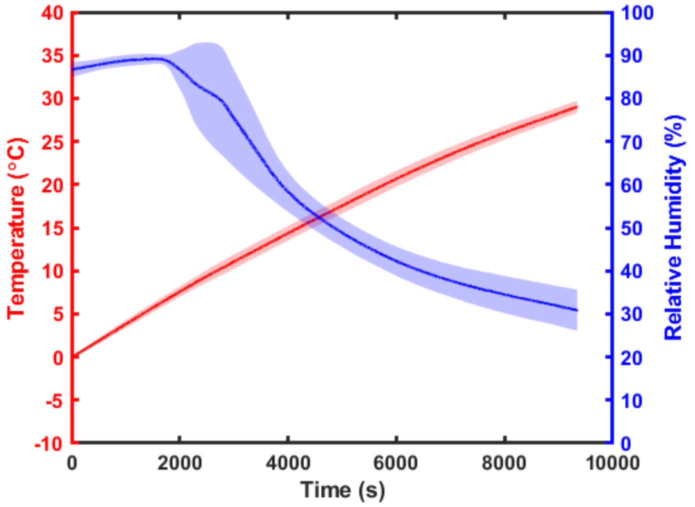

Fig. S16 | Average environmental parameters recorded over time once icepacks have been removed from the setup. The temperature and relative humidity data when exposing the tag to a gradual increase in temperature for experiments presented in **Fig. 4** was averaged with shaded area representing the standard deviation (n = 3).

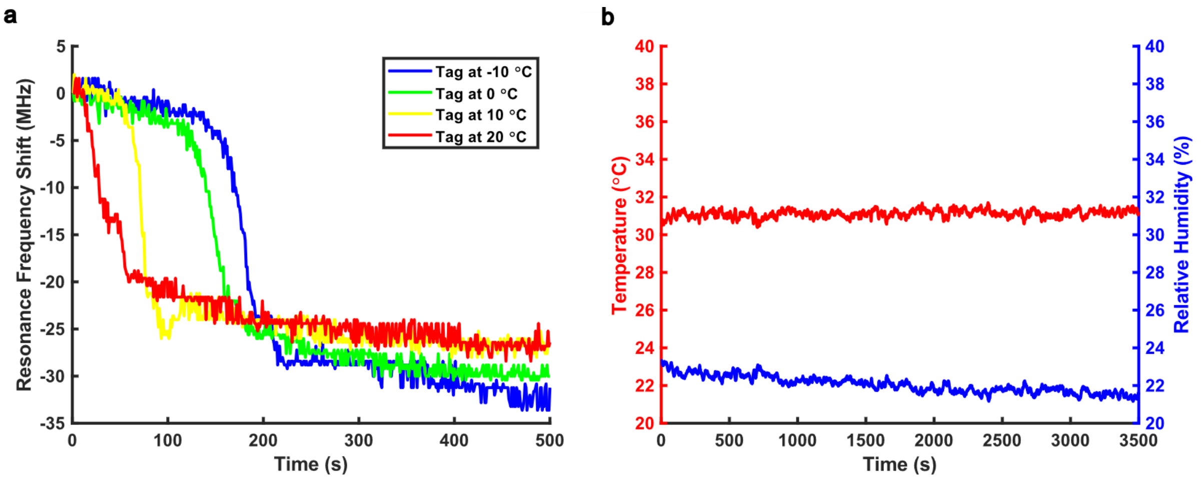

Fig. S17 | Time-temperature response of the tag with frozen coconut oil when exposed to air at 30 °C. a The frequency of resonance of the tag when suddenly exposed to 30 °C and 20% RH was measured over time for coconut oil in a frozen state at 4 initial temperatures of -10, 0, 10 and 20 °C. **b** The temperature and relative humidity of the air surrounding the tag is recorded during the various time-temperature experiments.

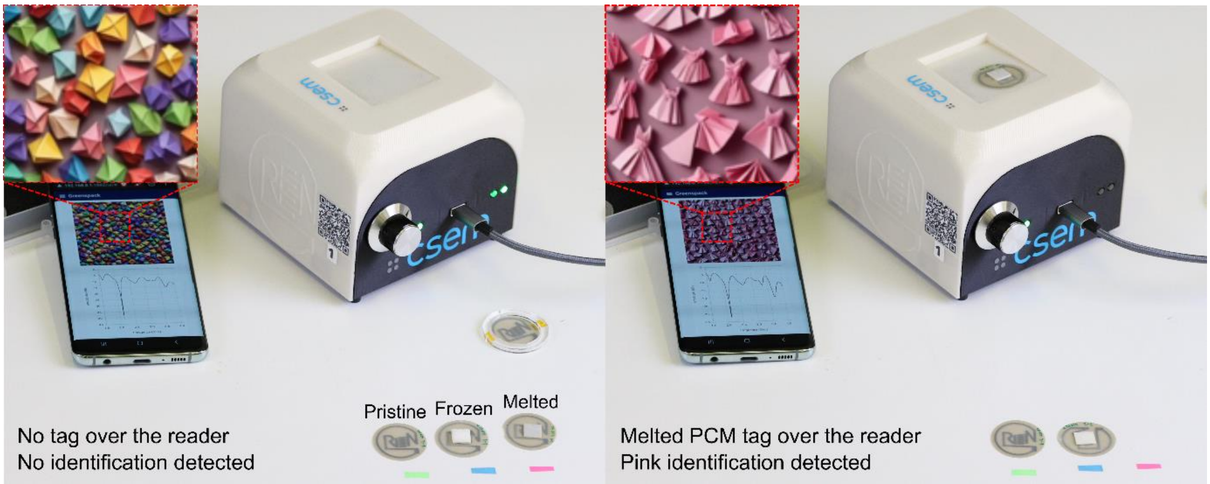

Fig. S18 | Wireless identification of the state of tag. The custom reader, interfaced with a smartphone, is used to identify the presence of a tag and differentiate its different states, displaying the S_{11} response with a corresponding colour (multi-colour when no tag is detected, green for pristine, blue for frozen coconut oil and pink for melted coconut oil). On the left image, no tag is placed over the reader, displaying a multi-colour pattern, while on the right, the reader detects the tag with the melted oil state, showing a pink pattern.
