# Supplementary material for: Ecoresorbable chipless temperature-responsive tag made from biodegradable materials for sustainable IoT
Source: Nat Commun. 2025 Nov 25;16:10478. doi: 10.1038/s41467-025-65458-9 (PMC12647737; doi:10.1038/s41467-025-65458-9)
Supplement: Supplementary file 2 — Description of Additional Supplementary Files [file 41467_2025_65458_MOESM2_ESM.pdf]

Description of Additional Supplementary Files

Supplementary Movie 1: Continuous recording of the melting of coconut oil over the tag. 64 µL of coconut oil is frozen in a cube and placed over the pristine tag. The S11 response of the tag is displayed from 2 °C to 30 °C with a picture of the tag taken every 30s while the temperature is monitored every second.

Supplementary Movie 2: Real-time detection of tags over custom-made portable reader. The portable reader configured with a smartphone detects three tags tested consecutively. The screen of the phone displays the S11 response and identification colour for each separate tag configuration. A multicolour pattern is chosen when no tag is positioned over the reader antenna, a green pattern is visible for pristine tag without PCM, a blue pattern is showing for a tag with paper element and frozen coconut oil, and a pink pattern is displayed for a tag with paper element soaked after melting of the PCM.
